# Immune mechanisms in the pathogenesis of endometriosis: a comprehensive analysis of the role of NK cells, cytokines, and extracellular vesicles/exosomes

**DOI:** 10.3389/fimmu.2026.1885409

**Published:** 2026-08-03

**Authors:** Ulrika Ottander, Emma Björk, Pernilla Israelsson, Lucia Mincheva-Nilsson

**Affiliations:** 1 Department of Clinical Sciences/Obstetrics and Gynecology, Umeå University, Umeå, Sweden; 2 Division of Obstetrics and Gynecology, Örnsköldsvik Hospital, Örnsköldsvik, Sweden; 3 Department of Diagnostics and Intervention/Oncology, Umeå University, Umeå, Sweden; 4 Department of Clinical Microbiology/Infection and Immunology, Umeå University, Umeå, Sweden

**Keywords:** endometriosis, immune surveillance, NK cells, NKG2D, MICA/B, FasL, cytokines, EVs/exosomes

## Abstract

**Introduction:**

Endometriosis is a chronic, estrogen-dependent inflammatory disorder affecting approximately 10% of women of reproductive age. Retrograde menstruation is widely accepted as a primary mechanism for ectopic endometrial seeding; however, only a subset of individuals develop the disease. This suggests additional pathogenic processes. Increasing evidence suggests impaired immune surveillance as a central factor enabling ectopic endometrial tissue to persist and expand. This review aims to explore immune-associated pathogenic mechanisms in endometriosis, focusing on the interplay between natural killer (NK) cells, cytokines, and extracellular vesicles (EVs).

**Methods:**

This study was conducted as a narrative review. Relevant PubMed studies addressing immune dysfunction in endometriosis were identified, with emphasis on the role of NK cells, cytokines, EVs, and EV-mediated signaling. All material chosen for referral in the review consists of published reports that were critically evaluated and discussed.

**Results:**

Endometriosis is associated with impaired immune surveillance, characterized by reduced NK-cell cytotoxicity, driven by altered receptor expression and a shift toward regulatory NK-cell subsets. A dysregulated cytokine milieu combines pro-inflammatory signals that promote lesion growth with immunosuppressive factors that inhibit immune clearance of ectopic tissue. Lesion-derived EVs further contribute the lesions’ survival by suppressing cytotoxic immune function, inducing apoptosis of activated immune cells and promoting inflammation and angiogenesis. The referred results highlight key immunological mechanisms underlying endometriosis.

**Discussion:**

This review presents an immune-based model in which NK-cell dysfunction, cytokine imbalance, and EV-mediated signaling cooperate to establish an immune-privileged microenvironment that promotes the survival and growth of ectopic endometrial tissue. The immune escape mechanism, described here, highlights potential targets as candidates to be tested for immunomodulatory therapies. We conclude that endometriosis should be considered a disorder fundamentally linked to mechanisms of immune dysregulation.

## Introduction

Endometriosis is a chronic, estrogen-dependent inflammatory disease characterized by the presence of endometrial-like tissue outside the uterine cavity (1). The prevalence is estimated to be 10% among women of reproductive age, with higher rates in those presenting with pelvic pain or infertility (2–4). Genetic predisposition is evident, with heritability estimated at approximately 50% (5). Several risk-enhancing and risk-reducing factors, summarized in **Table 1**, have been identified in endometriosis. The condition imposes a significant burden on healthcare systems and negatively impacts quality of life, including increased sickness absence and reduced income, underscoring broader life-course impact (6). Diagnosis of endometriosis is primarily based on clinical signs and physical examination supported by imaging and, when indicated, laparoscopy. Key diagnostic criteria include a history of progressive dysmenorrhea, non-menstrual pelvic pain, dyspareunia, dyschezia, dysuria, infertility, fatigue, and sometimes depression (4, 7). The cardinal symptom, pain, is nonspecific and may be attributed to a wide range of alternative causes and differential diagnoses, explaining the frequent diagnostic delays of several years (8, 9).

**Table 1:** Factors that enhance or reduce the risk of endometriosis.

Risk-enhancing factors	Reference	Risk-reducing factors	Reference
Early menarche	(182)	Parity	(183, 184)
Short menstrual cycles	(185)	Long duration of breastfeeding	(183, 184)
Nulliparity	(185)	Younger age at first pregnancy	(185)
Heavy menstrual bleeding	(186)		
Low body mass index	(187)		
Genital tract malformations	(188)		
Obstructive Müllerian anomalies	(189)		
Early-life soy exposure	(190)		
Exposure to EDCs*	(191)		

*EDCs , endocrine-disrupting chemicals.

Despite extensive research efforts, the etiology of endometriosis remains unknown. There are several competing theories regarding the origin of this multifactorial disease. The five most commonly discussed are retrograde menstruation, hematogenous or lymphatic dissemination, coelomic metaplasia, stem cell migration, and remains of the Müllerian ducts.

Retrograde menstruation is the most widely accepted mechanism for pelvic endometriosis (1). However, it is noteworthy that while retrograde menstruation is common, only a small subset of women develop endometriosis. This indicates a more complex etiology, where additional factors and mechanisms are involved. In health, well-established immune-surveillance mechanisms eliminate the displaced endometrial cells and restore homeostasis, protecting most women from the development of endometriosis. Logically, the inability to clear up ectopic endometriotic lesions should be associated with dysfunctional cells and responses, related to immune surveillance. Malfunctional natural killer (NK) cells were the first immune components to be consistently identified and suggested in the pathogenesis of this disease (10). Substantial evidence has shown that endometriosis arises from a convergence of interacting biological systems, where immune dysregulation amplifies and is amplified by other pathogenic pathways (11, 12). Aberrant immune activity, characterized by impaired clearance of ectopic endometrial cells, heightened inflammatory signaling, and altered macrophage and NK-cell function, creates a permissive microenvironment (11, 13) that supports endocrine disruption, including estrogen dominance and progesterone resistance (12, 14). These hormonal shifts, in turn, further skew immune responses toward chronic inflammation (14, 15). Parallel genetic variants and epigenetic modifications shape both immune behavior and hormone responsiveness, reinforcing lesion survival (16). Immune-derived cytokines also stimulate neuroangiogenic pathways, promoting nerve growth and vascularization that drive pain and lesion expansion (17, 18). Finally, immune-mediated inflammation may influence the recruitment and differentiation of stem-cell–related progenitors, contributing to the heterogeneity and resilience of lesions (16). Together, these intertwined mechanisms form a self-sustaining network that underlies the chronic, multifactorial nature of endometriosis (11).

This review concentrates specifically on the dysregulated immune mechanisms that enable immune tolerance of ectopic endometrial-like tissue. We summarize current evidence supporting the view that endometriosis is a disorder driven by immune dysfunction. We focus on three major immune components, NK cells, cytokines, and extracellular vesicles (EVs), and examine their roles in disease development and pathogenesis. By integrating findings from our own work with those of others, we propose a schematic model illustrating how these components interact to protect endometriotic lesions from immune recognition and destruction, thereby promoting disease development.

## Methods

This study was conducted as a narrative review aimed at summarizing and synthesizing current knowledge on the topic. Relevant literature addressing the roles of NK cells, cytokines, and EVs in endometriosis was identified through PubMed searches using terms related to endometriosis and these immune-system components. Reference lists of relevant articles were also screened to identify additional studies. All studies selected for inclusion were critically evaluated and discussed.

## Natural killer cells

### General definition and properties

NK cells are cytotoxic lymphocytes of the innate immune system that identify and kill infected, stressed, or pathologically transformed cells. In peripheral blood, NK cells represent approximately 5–15% of all circulating leukocytes. Their killing ability is initiated either directly, through engagement of NK-cell-specific receptors, or indirectly, via an antibody-dependent cell-mediated cytotoxicity (ADCC) pathway. Unlike adaptive T and B cells, NK cells are not dependent on antigen presentation by the major histocompatibility complex/human leukocyte antigen (MHC/HLA), and their receptors are not antigen-specific. Instead, they react to foreign, missing, or altered MHC/HLA patterns through activating and inhibitory receptors, via a mechanism explained in the “missing self” hypothesis (19). NKG2D is the main activating cytotoxic receptor that plays a critical role in immune surveillance (20). It interacts with multiple stress-induced ligands, with the two major groups in humans being the MHC class I chain-related antigens A and B (MICA/B), and UL16-binding proteins (ULBP) 1–6 (21).

The two subsets of NK cells in humans are CD56^bright^/CD16^+^ cells and CD56^dim^/CD16^-^ cells, classified by the density of the CD56 molecule’s cell-surface expression and the expression of the Fcγ receptor CD16 (22). The CD56^dim^/CD16^+^ subset exhibits a higher intrinsic cytotoxic potential and, besides direct killing, can also kill target cells by an ADCC pathway due to the expression of CD16. The CD56^bright^/CD16^-^ cells primarily produce cytokines, exhibit low cytotoxicity, and have a regulatory function (23). Both CD56^bright^/CD16^+^ and CD56^dim^/CD16^-^ express the activating NKG2D receptor (24). “Conventional” NK cells, i.e. CD56^dim^/CD16^+^, predominate in the peripheral blood, while the CD56^bright^/CD16^-^ NK cells in the circulation are very low/absent (25). Instead, the CD56^bright^/CD16^-^ NK cells comprise tissue-resident NK cells, localized in organs such as the thymus, liver, and uterine mucosa (26). A comparison between CD56^dim^/CD16^+^ and CD56^bright^/CD16^-^ NK cells is presented in **Table 2**.

**Table 2:** Comparison of CD56^dim^/CD16^+^ and CD56^bright^/CD16^-^ NK cells.

Feature	CD56^dim^/CD16+ NK cells	CD56^bright^/CD16- NK cells
Localization	Predominantly in peripheral blood	In the thymus and secondary lymphoid organs In the intestine, liver, and uterine mucosa
Proportion in peripheral blood	5–15% of PBMC	0–<2% of PBMC
Proportion in total peripheral NK cells	~90%	0~10%
Cytokine production	Low	High
Cytokines	IFNγ, TNFα	TGFβ1, IL-10, IL-13, IFNγ, TNFα, GM-CSF*
Cytotoxic capacity	Strong Efficient ADCC**	Weak
Perforin and granzyme content	High	Low
Regulatory capacity	None	High
Receptor profile	More inhibitory KIRs** *	Fewer KIRs High CD94/NKG2A Strong sensitivity to IL-2 and IL-21
CD16 (Fcγ receptor) expression	High Efficient ADCC	None Unable to perform ADCC
Functional role	Elimination of infected, stressed, or transformed cells	Immunoregulation Angiogenesis Tissue remodeling

*GM-CSF, Granulocyte-macrophage colony stimulating factor.

**ADCC, Antibody-dependent cell-mediated cytotoxicity.

***KIR, Killer-cell immunoglobulin-like receptor.

Within the uterine mucosa, the CD56^bright^/CD16^-^ tissue-resident NK cells are a unique subset, known as uterine NK (uNK) cells; these are further classified into endometrial NK (eNK) and decidual NK (dNK) subsets. They produce large quantities of the regulatory cytokines TGFβ1 and IL-10, exhibit limited cytotoxic activity, and play a crucial role in promoting immune tolerance. Additionally, they participate in stimulating neo-angiogenesis, and facilitating tissue construction and remodeling, processes that are essential in the menstrual cycle and placenta formation during pregnancy (27, 28). Uterine NK cells represent approximately 20% of lymphocytes in the proliferative endometrium, increasing to 50% in the secretory phase and up to 70–80% in early gestation (25).

### NK cells in endometriosis

NK-cell dysfunction is a key immunological defect in endometriosis. The NK cells fail to clear endometriotic lesions due to reduced numbers and impaired function. Abnormal receptor–ligand interactions and increased levels of immunosuppressive cytokines are suggested as an explanation for NK-cell dysfunction. The impairment of NK cells, which is primarily extrinsic, is driven by the inflammatory and immunosuppressive microenvironment (12). Secondarily, there are also intrinsic factors such as reduced expression of the cytotoxic effector genes PRF1 (perforin) and GNLY (granulysin) within the NK cells themselves that could weaken the strength of the cytotoxic hit (29). Abnormalities in NK-cell function correlate with disease severity (30). NK-cell levels are lower in endometriotic lesions than in the endometrium of both endometriosis patients and healthy controls (31, 32). NK cells in the peritoneal fluid of endometriosis patients are also reduced in number and exhibit diminished cytotoxic activity, thereby facilitating immune escape of the endometriotic tissue at ectopic sites (33). Aberrant distribution of NK-cell subsets has been reported with higher levels of CD56^bright^/CD16^-^ NK cells, which secrete large amounts of the immunoregulatory cytokines IL-10 and TGFβ1 in peritoneal fluid and peripheral blood (34–36). TGFβ1 diminishes NK-cell cytotoxicity and suppresses the expression of the activating receptor NKG2D (37). The reduced cytotoxic function observed in NK cells partially depends on the presence of increased levels of CD56^bright^/CD16^-^ eNK cells, both systemically and locally. We recently reported increased numbers of CD56^bright^/CD16^-^ NK cells in the peripheral blood of untreated endometriosis patients, probably due to a leakage of these cells from the endometriotic lesions into the circulation (38). Their numbers were normalized after surgical and hormonal treatment. Based on these studies (34–38), we suggest that part of the observed NK-cell dysfunction might be due to increased levels of circulatory CD56^bright^/CD16^-^ NK cells. Their low cytotoxic capacity and high production of immunosuppressive cytokines promote the survival and proliferation of endometriotic tissue at ectopic sites (38).

Another reason for the decrease in NK-cell cytotoxicity is a strong association with a dysregulated balance between activating and inhibitory receptors (39). A decreased expression of activating receptors and an increased presence of inhibitory receptors on these NK cells have been described (33, 40–43), where specifically the activating NKG2D receptor was downregulated. The downregulation of NKG2D limits NK-cell recognition of ectopic endometrial cells and suppresses perforin/granzyme release, thus favoring immune escape (37). Increased shedding of the cognate ligands to the activating NKG2D receptor has been suggested as a mechanism that downregulates the expression of the latter, supported by a report showing upregulated levels of soluble NKG2D ligands MICA/B. MICA/B levels in peritoneal fluid have also been found to have a positive association with disease severity (44). In a recent publication, we described another mechanism of NKG2D downregulation, mediated by exosomes. We demonstrated that endometriotic lesions secrete exosomes that express the NKG2D ligands MICA/B and ULBP 1–3 on their surface. These exosomes downregulate the NKG2D receptors on NK cells, thereby impairing NK-cell cytotoxicity (45). The increased levels of soluble MICA/B reported by González-Foruria et al. (44) are likely exosome-associated ligands that suppress NK-cell activation and contribute to impaired immunosurveillance (46, 47). In another report (48), peritoneal NK cells in endometriosis expressed elevated levels of the inhibitory receptor NKG2A. This shift toward inhibitory signaling was linked to disease progression. Activation of NKG2A reduced degranulation and IFNγ secretion, and thus the overall cytotoxic capacity (39, 49). In addition, dysregulation of adhesion molecules further undermines NK-cell function (50). Besides exosome-mediated NKG2D downregulation, we observed that exosomes secreted by endometriotic tissue express Fas ligand (FasL) and TNF-Related Apoptosis-Inducing Ligand (TRAIL), which induced apoptosis of activated immune cells promoting immune privilege of the lesions (45). This finding could explain the reduced NK-cell numbers frequently reported in endometriosis.

## Cytokines

### General description of cytokines and their functions

#### Common features

Cytokines are small, secreted proteins/peptides that enable intercellular communication and regulate immunity, inflammation, hematopoiesis, and homeostasis. They can be secreted by almost all cells, depending on the microenvironment, and act on their target cells, locally or distantly, through specific receptors. Stimulation by cytokines is considered to be autocrine when the secreting cell is stimulating itself, paracrine when neighboring cells are stimulated, and endocrine when distant cells are stimulated. Cytokines can be produced by many cells, such as epithelial and endothelial cells and fibroblasts, but the cells of the immune system are some of the most important producers of cytokines. Cytokines are grouped into families such as interleukins, interferons, tumor necrosis factors, and chemokines (51).

#### Cytokines regulate immune responses

Cytokines play a pivotal role in the function of the immune system through the initiation and regulation of cellular and humoral immune responses, promotion of inflammation, and maintenance of immune surveillance and homeostasis. Depending on the local microenvironment, activated T helper- and other cells are induced to produce various cytokines that can be grouped into cytokine responses with specific functions (**Table 3**) designated as follows: 1) T helper (Th) 1 response, including IFNγ, IL-12, and IL-15, which promotes cytotoxicity and stimulates MHC class I and II expression; 2) Th2 response, including IL-4 and IL-13, which supports humoral immunity; 3) proinflammatory response, including IL-1β, IL-6, IL-8, TNFα, and IL-17, which initiates and amplifies inflammation; and 4) T regulatory response, driven by TGFβ1 and IL-10, which induces immune suppression, maintains immune tolerance, and protects homeostasis (52).

**Table 3:** Regulation of immune responses by cytokines.

Cytokine response	Involved cytokines	Immune response
T helper 1	IFNγ*, IL-12, IL-15	Cellular response –cytotoxicity
T helper 2	IL-4*, IL-13	Humoral response – antibody production IgE-mediated allergic response (adverse reaction)
Proinflammatory	TNFα*, IL-6, IL1β*, IL-8, IL-17*	Inflammation
Regulatory	IL-10*, TGFβ1*	Immune suppression

*Index cytokine.

### Cytokines in endometriosis

#### Methodological considerations

The vigorous secretion of cytokines in endometriosis induces a variety of immune mechanisms that act in concert to impair the clearance of ectopic endometrial tissue. However, studies often report contradictory results regarding cytokine levels and responses. There may be several reasons for this, including differences in study design, sample type (e.g., peritoneal fluid, eutopic or ectopic endometrial tissue, endometrioma tissue, or cyst fluid), disease stage, and hormonal or pharmacological influences. Discrepancies in results may also arise from differences in methodology and sample handling. Cytokines can be analyzed using various methods: ELISA (most commonly used), immunoflow cytometry, Western blot, quantitative RT-PCR, immunohistochemical intracellular staining, and *in-situ* hybridization. Notably, cytokines are small proteins/peptides that are highly susceptible to degradation, which can affect the reliability of analytical results. Appropriate measures to prevent degradation should be taken during blood collection, transport, and storage for future cytokine analyses. Overall, these sources of variability underscore the complexity of cytokine assessment in endometriosis and complicate the interpretation of results. To illustrate this, **Table 4** summarizes results from published reports on cytokines and their proposed roles in endometriosis in a more detailed manner.

**Table 4:** Summary of published reports on cytokine production and role in endometriosis.

Response and cytokine* (52)	Levels in endometriosis patients	Function in immune evasion	Biomarker potential
Promoting cytotoxic immune response (Th1)			
IFNγ	• ↑ PF (flow cytometry) (84) • ↓ PF (ELISA) (101) • ↑ PBMC (RT-qPCR) (62) • ↓ Endometriotic tissue (RT-qPCR) (62)	• ↑ Intercellular adhesion molecule-1 expression (104), potentially facilitating invasion of ectopic endometrial tissue • Normally inhibits proliferation and induces apoptosis, but effect blocked by anti-apoptotic proteins (105)	• No significant difference in serum levels between endometriosis patients and healthy women (192)
IL-12	• ↑ PF (ELISA) (106, 107) • No difference in PF (ELISA) (109, 110). • ↑ Serum, in advanced compared to earlier stages (ELISA) (106)	• IL-12 treatment enhances NK-cell recognition of endometrial cells (109) • Peritoneally administered IL-12 reduces lesions in mouse model (108)	• Unclear
IL-15	• ↑ PF and ectopic endometrium (RT-qPCR, ELISA, IHC) (111, 112) • ↓ PF in advanced stages (ELISA) (113)	• ↓ Apoptosis • ↑ Invasiveness and proliferation of ESCs • ↓ NK-cell cytotoxicity markers (CD16, Granzyme B, IFNγ, NKG2D) (114)	• Unclear
Promoting humoral immune response (Th2)			
IL-4	• ↑ Peripheral blood and endometrial tissue (RT-qPCR) (86) • ↑ Serum and PF (ELISA) (117)	• ↑ EMSC proliferation (73) • ↑ Fibrosis markers (periostin and TCF21) (119)	• Serum (117, 193) and PF (117) can be used to discriminate between endometriosis patients and controls
IL-13	• ↑ PF and ectopic endometrium (RT-qPCR, ELISA, IHC) (111, 116) • ↓ PF (multiplex immunoassay) (118)	• ↑ Fibrosis markers (periostin and TCF21) (119)	• Unclear
Proinflammatory response			
IL-6	• ↑ PF (ELISA) (55, 59, 60) (multiplex immunoassay) (54) • ↑ Serum (ELISA) (55, 60, 61) • ↑ Endometriotic tissue and endometrium (ELISA) (58), (RT-qPCR) (62)	• Suppresses NK-cell function (78) • ↑ Haptoglobin ➔ reducing phagocytosis (194)	• Correlates with stage (60) • Serum levels discriminate between endometriosis patients and controls (61) and were predictive of endometriosis (57)
TNFα	• ↑ PF (ELISA) (61, 69) (multiplex immunoassay) (54) • ↑ Serum (ELISA) (69) • ↑ PBMC (RT-qPCR) (62)	• Induces IL-8 expression (72) • Stimulates matrix metalloproteinases ➔ matrix remodeling (71) • Promotes endometrial cell proliferation (75)	• PF levels can be used to discriminate between endometriosis patients and controls (61)
IL-1β	• ↑ PF (EL-4 assay) (53) (multiplex immunoassay) (54) (ELISA) (55) • ↑ Cervico-vaginal fluid (56) • ↑ Serum (Multiplex Cytokine Panel) (57) (ELISA) (55) • ↑ Endometriotic tissue (ELISA) (58) • ↑ PBMC (RT-qPCR) (62)	• ↑ IL-8 ➔ immune cell recruitment and angiogenesis (74) • ↑ Matrix metalloproteinases ➔ matrix remodeling (71)	• Cervico-vaginal fluid levels can be used to discriminate between endometriosis patients and controls (56) • Serum levels predictive of endometriosis (57)
IL-8	• ↑ Eutopic endometrium (gene expression analyses) (195), (RT-qPCR) (62) • ↑ PF (ELISA) (63, 64) (multiplex immunoassay) (54) • ↑ Cystic fluid of endometriomas (65)	• ↑ EMSC proliferation (72) • ↑ Cell number and DNA synthesis in ESCs and EMSCs (63) • ↑ Angiogenic activity (64) • ↑ Adhesion of endometrial stromal cells to the extracellular matrix (70)	• Correlates with severity (63)
IL-17	• ↑ PBMC (flow cytometry) (66) and PF (ELISA) (67) of IL-17-producing cells • ↑ Eutopic and ectopic endometrial tissue (IHC) (66) • ↑ PF (67), and PF of patients with minimal or mild endometriosis compared to moderate or severe or no endometriosis (ELISA) (68) of IL-17 • ↑ Plasma of endometriosis patients compared to controls (multiplex assay) (79)	• ↑ Survival and migration of endometrial cells (67) • ↑ Expression of anti-apoptotic genes (67) • ↓ Reduced NK cell-mediated cytotoxicity (67) • ↑ HLA-G expression on endometrial cells (67) • ↑ IL-8 secretion, expression of cyclooxygenase-2, and proliferation of ESCs (76)	• Unclear
Immunoregulatory (Treg cells, Th3, Tr1)			
TGFβ1	• ↑ Serum and PF (ELISA) (55, 69, 80–82), • ↑ Endometriotic bowel lesions (RT-qPCR) (83), • ↑ Eutopic endometrium (RT-qPCR) (62) • ↑ PBMC (RT-qPCR) (62)	• ↓ NKG2D and cytotoxic function of NK cells (81, 87, 196) • ↑ Attachment and invasion (92, 93) • ↑ Fibrosis (98)	• Serum levels can be used to discriminate between early-stage endometriosis and controls (197)
IL-10	• ↑ PF (flow cytometry) (84) (multiplex immunoassay) (54) (ELISA) (55) • ↑ Serum (ELISA) (55, 85) • ↑ Peripheral blood lymphocytes (RT-qPCR) (86) • ↑ Endometriotic bowel lesions (RT-qPCR) (83) • ↑ Eutopic endometrium (RT-qPCR) (62). • ↑ PBMC (RT-qPCR) (62)	• Conditions dendritic cells ➔ ↑ IL-10 and ↓ IL-12 (85) • ↑ Endometriotic growth (85) • Promotes pro-fibrotic phenotype (99)	• Serum levels can be used to discriminate between endometriosis patients and controls (193)
IL-2	• ↓ PF (ELISA) (117). • ↑ PF in advanced stages (flow cytometry) (91) • ↑ Endometrium and endometriotic lesions (RT-qPCR) (62, 198)	• IL-2–activated NK cells induce apoptosis in and reduce migration and invasion of ectopic ESCs (199) • Recombinant IL-2 reduces implant size in rats (200)	• PF levels discriminate between endometriosis and healthy controls (117)

Th, T helper; IFN, interferon; PF, peritoneal fluid; ELISA, enzyme-linked immunosorbent assay; IL, interleukin; NK, natural killer; RT-qPCR, reverse transcription quantitative polymerase chain reaction; IHC, immunohistochemistry; ESCs, endometrial stromal cells; EMSCs, endometriotic stromal cells; HLA, human leukocyte antigen; Treg, Tregulatory; Th3, T helper 3; Tr1, Tregulatory 1.

*Cytokines may exhibit other functions.

#### Cytokines in endometriosis

Endometriosis is strongly driven by inflammation, with elevated levels of cytokines such as IL-1β (53–58), IL-6 (54, 55, 58–62), IL-8 (54, 62–65), IL-17 (66–68), and TNFα (54, 61, 69) reported in peritoneal fluid, endometriotic lesions, and serum from affected patients. These cytokines promote key pathogenic processes, including adhesion, proliferation, invasion, and angiogenesis. IL-8 promotes the adhesion of endometrial stromal cells to the extracellular matrix (70), while TNFα and IL-1β (71) stimulate the expression of matrix metalloproteases (MMPs), which support tissue remodeling and potentially endometrial cell implantation. IL-8, IL-17, and TNFα also enhance the proliferation of endometriotic cells, which is amplified through a positive feedback loop in which TNFα, IL-17, and IL-1β upregulate IL-8 secretion (72–74). Similarly, Iwabe et al. reported elevated IL-8 concentrations in the peritoneal fluid of endometriosis patients, which increased cell number and DNA synthesis in both endometrial and endometriotic stromal cells (63). Recombinant TNFα further stimulated the proliferation of eutopic and ectopic endometrial cells from women with endometriosis, but inhibited proliferation of eutopic endometrial cells from controls, suggesting dysregulated TNFα signaling in the disease (75). IL-17 has similarly been shown to promote proliferation of endometriotic stromal cells (73), enhance endometrial cell survival, and increase the expression of anti-apoptotic genes (67). IL-17 upregulates cyclooxygenase-2 (76), a key enzyme involved in the prostaglandin pathway and an important mediator of inflammation. Kang et al. demonstrated that recombinant IL-17A reduces NK cell-mediated cytotoxicity, induces human leukocyte antigen (HLA)-G expression on endometrial cells, and promotes the migration of the latter (67). Since HLA-G is a critical immunomodulator in maternal-fetal tolerance that suppresses both decidual and peripheral NK cells (77), these findings might support an immunosuppressive role for IL-17 in endometriosis. Elevated IL-6 levels in the peritoneal fluid of endometriosis patients correlate with reduced granzyme B and perforin expression, impairing NK-cell cytotoxicity (78). IL-6 also interferes with the clearance of endometriotic lesions by inducing release of a haptoglobin variant from these lesions, which diminishes macrophage phagocytic activity and further increases IL-6 production (78). Increased IL-8 levels in peritoneal fluid are associated with enhanced angiogenic activity (64). IL-1β and IL-17 further promote angiogenesis by simulating IL-8 and VEGF secretion (74, 76, 79).

A compensatory immunosuppressive response accompanies the proinflammatory milieu in endometriosis, largely driven by regulatory T (Treg) cells and the cytokines TGFβ1 (55, 62, 69, 80–83) and IL-10 (54, 55, 62, 83–86), which are predominantly elevated in peritoneal fluid, serum, and endometriotic lesions of affected patients. TGFβ1 downregulates the NKG2D receptor on NK cells (81) and induces the expression of indoleamine 2,3-dioxygenase (IDO), which is associated with further reduction in NKG2D expression (87), collectively impairing NK-cell cytotoxicity. Increased numbers of Forkhead box P3 protein-positive (FOXP3^+^) Tregs in peritoneal fluid (88) and endometriotic lesions correlate with higher TGFβ1 levels (89). A recent study confirmed an increased proportion of Tregs in the peritoneal fluid of endometriosis patients, accompanied by higher concentrations of TGFβ1 and IL-10 (90), suggesting a self-reinforcing loop for Treg differentiation. The same study demonstrated that peritoneal fluid acts as a chemoattractant for Tregs and CD4^+^ Th cells, supporting a Th2-skewed immune response (90). IL-10 and TGFβ1 produced by endometrial stromal cells from endometriosis patients downregulate NKG2D and CD16, reduce IFNγ production, and diminish NK-cell viability and cytotoxicity. TGFβ1 activates macrophages, inducing IL-2 secretion, essential for Treg maintenance (91). Beyond immune modulation, TGFβ1 promotes attachment and invasion of ectopic endometrial cells by upregulating integrins, plasminogen activator inhibitor-1, and colony-stimulating factor-1 receptor (92–94). Hypoxia further amplifies this adhesive capacity through a positive feedback loop that enhances TGFβ1 signaling (95). It has been shown in mouse models that IL-10 inhibition reduces lesion size, whereas IL-10 administration promotes lesion growth (85). Both cytokines contribute to fibrosis, which is a hallmark of endometriosis (96). TGFβ1 induces fibroblast-to-myofibroblast transdifferentiation and increases MMP secretion (97) and epithelial-mesenchymal transition, leading to smooth muscle metaplasia and fibrosis (98). IL-10 promotes a pro-fibrotic phenotype specifically in endometriotic stromal cells (99). Additionally, both cytokines stimulate angiogenesis (85, 100), supporting lesion survival and expansion.

Studies of IFNγ in endometriosis show inconsistent results, with reports of both increased (84) and decreased levels (101). Normally, IFNγ is produced by uNK cells during implantation (102). A transient serum increase after blastocyst transfer in successful *in-vitro* fertilization cycles suggests a physiological role in early pregnancy (103). In endometriosis, elevated IFNγ in peritoneal fluid has been linked to increased Intercellular Adhesion Molecule-1 (ICAM-1) expression, potentially facilitating implantation of ectopic endometrial tissue (104). Although endometriotic stromal cells express IFNγ receptors, IFNγ does not suppress their proliferation or induce apoptosis, likely due to the upregulation of anti-apoptotic factors (105). Thus, IFNγ may enhance the invasiveness of endometriotic cells without exerting a cytotoxic effect. The conflicting results reported for IFNγ in patients with endometriosis suggest context-dependent dysregulation, rather than consistent upregulation or downregulation. The upstream regulator IL-12 shows similarly inconsistent patterns. Reported elevated levels, particularly in advanced disease (106, 107), suggest a Th1-skewed immune response in later disease stages. Functionally, IL-12 has been shown to reduce lesion size in animal models (108) and to enhance NK cell-mediated recognition of endometrial cells *in vitro* (109), indicating a potentially protective role. Other studies report no differences between patients and controls nor across disease stages (109, 110). Further research is needed to clarify the role of both IFNγ and IL-12. IL-15 is elevated in the peritoneal fluid and ectopic lesions of endometriosis patients (111, 112), although one study reported reduced levels in advanced disease (113). IL-15 promotes endometrial stromal-cell proliferation and invasiveness, while suppressing NK-cell cytotoxicity by downregulating CD16, Granzyme B, IFNγ, and NKG2D (114). IL-15 is a central regulator of NK-cell development and function, and its activity in the endometrium is normally tightly controlled. Chronic exposure *in vitro* induces a negative feedback loop, reflecting the impaired NK-cell phenotype observed in endometriosis patients (115).

In endometriosis there is a Th2-skewed immune profile, with elevated IL-4 and IL-13 in peritoneal fluid, serum, and ectopic lesions (84, 86, 111, 116, 117). This Th2 bias creates an environment that promotes tissue repair, fibrosis, and reduced cytotoxic activity, all supporting lesion survival. However, one study reported lower IL-13 levels in the peritoneal fluid of endometriosis patients compared to controls (118), although it should be noted that both groups consisted of infertility patients, with or without endometriosis. This suggests that there may be other biological variations present in the control group, making the interpretation of the results more difficult. Ganieva et al. demonstrated that IL-4 and IL-13 upregulate fibrosis-associated markers (119). In addition to its pro-fibrotic effects, IL-4, synergistically with TNFα, directly stimulates the proliferation of endometriotic stromal cells (73). Both cytokines also induce the polarization to M2 macrophages (120), a phenotype associated with tissue remodeling, angiogenesis, and fibrosis. Consistently, patients show increased M2-like macrophages as well as elevated Th2 and Treg populations in peritoneal fluid (121). Overall, IL-4 and IL-13 promote stromal proliferation, fibrogenesis, M2 polarization, and structural remodeling, helping maintain the microenvironment that enables endometriotic lesions to persist.

A shortcoming of these studies is that they were conducted on a limited number of cytokines, often 2–4 per study, which does not reflect the broader overall picture of the cytokine network operating in endometriosis. To obtain a more comprehensive overview of the cytokine network and responses in endometriosis, we performed a simultaneous RT-qPCR analysis, specifically quantifying mRNA levels of 11 cytokines in endometriotic lesions, eutopic endometria, and peripheral blood mononuclear cells from endometriosis patients and age-matched controls. The 11 cytokines were chosen to represent the four major cytokine response patterns/profiles, shown in **Table 3** (62). Due to its high sensitivity and reproducibility, RT-qPCR-based determination of cytokine mRNA profiles is recommended for comparing patient groups and exploring mechanistic pathways (122). Our analysis (62) suggests a coordinated inflammatory and regulatory response; however, these findings reflect our specific methodological approach and should be interpreted in conjunction with the broader, and sometimes heterogeneous, literature. We found a pronounced systemic and local inflammatory response in patients with endometriosis, without the expected upregulation of IFNγ. Instead, elevated TGFβ1 and IL-10 expression suggested an enhanced regulatory response. Notably, this response was accompanied by an increased IL-2 mRNA expression in the endometriotic lesions only, indicating local induction and/or proliferation of inducible Treg cells. This finding was confirmed by immunohistochemical staining that revealed an abundance of regulatory cells in the endometriotic tissue. Overall, the cytokine mRNA profiles reflected combined inflammation, immunosuppression, and active induced Treg (iTreg) proliferation, processes that promote immune evasion and persistence of ectopic lesions (122). In conclusion, our findings and those of others demonstrate a consistent pattern of cytokine network dysregulation contributing to disease progression.

## Extracellular vesicles in endometriosis

### Definition, general description, and properties of extracellular vesicles

The International Society for Extracellular Vesicle Research (ISEV) defines EVs, including all naturally occurring or engineered vesicles, as “particles that are released from cells into the extracellular space, are delimited by a lipid bilayer and cannot replicate on their own” (123). EVs can be broadly divided by size into small EVs (< 200 nm in size) and large EVs (>200 nm in size) (123). Exosomes, microvesicles/microparticles, and apoptotic bodies/blebs comprise three main groups of EVs derived from eukaryotic cells. A brief overview of some of their characteristics and roles in health and disease (124) are summarized in **Table 5**.

**Table 5:** Physical characteristics, presence, and roles of the three main types of EVs produced by eukaryotic cells in health and disease.

Characteristics	Small EVs/exosomes	Larger/medium EVs shed microvesicles microparticles	Apoptotic bodies apoptotic blebs
Origin	The endosomal compartment of eukaryotic cells	Plasma membrane	Fragments of dying cells
Produced through	Inward budding in endosomes by ESCRT* and ceramide-dependent pathways	Budding from the plasma membrane by fragmentation and detachment from the cytoskeleton	Fragmentation of dying cells by actin-myosin fibril contraction, release of lactate dehydrogenase
Size	30–150 nm	0.1–2 μm	0.05–5 μm
Shape and appearance in TEM**	Cup-shaped Electron-translucent	Various shapes: round, elongated, cylinder-like Electron-dense and/or electron-translucent	Irregular and heterogeneous Electron-dense and/or electron-translucent
Specific marker(s) for identification	Tetraspanins (CD63, CD9, CD81), ESCRT complex members (Alix, TSG101)	Integrins, selectins, CD40, and others, depending on the cell type	Histones, DNA, caspases
Release/ secretion	Exocytosis by fusion of MBV*** with the plasma membrane	Plasma membrane blebbing	Plasma membrane blebbing and cellular fragmentation
Cells, producing EVs	Blood cells: reticulocytes, platelets, mast cells, immune cells (T and B lymphocytes, macrophages, monocytes, and dendritic cells). Most epithelial cells (bronchial epithelium, enterocytes, uroepithelium), Hepatocytes, neurons, glial cells, syncytiotrophoblast. A vast range of tumor cells		
EV presence in biofluids	In vivo: blood and other biofluids: ocular effluent, aqueous humor, cerebrospinal fluid, nasal and bronchial secretions, saliva, bile, synovial fluid, breast milk, urine, amniotic fluid, semen, and various pathological effusions such as ascites, pulmonary effusions, and cyst fluids. In vitro: secreted in the supernatant of cell and organ cultures.		
EV role in biological processes	Small EVs/exosomes: pregnancy, embryogenesis, differentiation, development, intercellular signaling, communication by proxy, delivery of proteins to the plasma membrane and cytoplasm of recipient cells, initiation of pro- or anti-apoptotic pathways, antigen presentation, immune regulation, and reprogramming of cells via delivery of bioactive nucleic acids (131, 201). Large EVs: intercellular communication, waste removal, signal transduction, wound healing, tissue regeneration, protecting of homeostasis		
EVs’ role in diseases	Small EVs/exosomes: cancer (establishment, growth, and metastasis), pre-eclampsia, endometriosis, polycystic ovarian syndrome, cardiac diseases, rheumatoid arthritis, and allergy (135, 140–143). Large EVs: promote inflammation, pro-inflammatory cytokine production, neo-angiogenesis, metastasis, and coagulation. Associated with diseases such as cardiovascular hypertension, cancer, autoimmunity disorders, and pre-eclampsia.		

*ESCRT , endosomal sorting complexes required for transport.

**TEM , transmission electron microscopy

***MVB, microvesicular bodies of the endosomal complex

Exosomes (30–150/200 nm), the smallest EVs secreted by eukaryotic cells, comprise a unique class of EV, and originate in the endosomal cell compartment. They are produced by inward membrane budding, and are stored within and subsequently secreted by microvesicular bodies (MVBs) (125). Their membrane, distinctly different from the plasma membrane of eukatyotic cells, is enriched with tetraspanins, cholesterol, and sphingolipids. It is robust and resistant to detergents and mechanical manipulation such as prolonged high-speed centrifugation and freeze-thawing. Consequently, the content/cargo carried inside exosomes is well-protected. While their size is typically 30–150 nm, as measured by nanoparticle tracking instruments such as ZetaView^®^ and Nanosight^®^, their upper size limit when measured by electron microscopy (EM) can be no larger than 100 nm, which is an artefact due to shrinkage. The characteristic EM image of an exosome shows a cup-shaped morphology, which is an artefact caused by membrane collapse during ultracentrifugation. The exosomal composition reflects the state of the parental cells, and includes proteins and nucleic acids such as DNA, microRNA (miRNA), and long non-coding RNA (lncRNA). In humans, small EVs/exosomes are produced and secreted by virtually all types of nucleated cells. Their physiological and pathogenic roles depend on the nature of the donor and recipient cells, and the environmental context (126–130). The majority (approximately 80%) of small EVs in the peripheral blood of healthy subjects are derived from circulating platelets. Because EVs function as means of intercellular communication and thus are destined for uptake by recipient cells, they are short-lived, with a circulatory half-life of less than five minutes (131).

In contrast, medium-to-large (0.2–1 μm) EVs, also known as microvesicles, microparticles, or ectosomes, are produced by membrane blebbing, which occurs when proteolytic enzymes cleave the cytoskeleton from the plasma membrane (126, 130, 132–134). Normal cellular turnover as well as various stimuli – including cell activation, inflammation, hypoxia, oxidative stress, complement activation, and cell damage – can induce membrane blebbing and the release of these microvesicles (135).

The third type of large (0.5–5 μm) EVs, known as apoptotic bodies or blebs, are produced by the fragmentation of dying cells undergoing apoptosis. They contain cellular organelles, caspases, and other cytoplasmic components, and alongside immune cells participate in the clearance of inflammatory products (126).

Regarding the effect on the immune system, small EVs (i.e. exosomes) can be either immunoactivating or immunosuppressive (136). Generally, exosomes, which are produced by antigen-presenting cells (APCs), such as macrophages, monocytes, dendritic cells, and B cells, are immunoactivating. They can upregulate immune cells directly, acting as antigen presenters by proxy, or indirectly, through uptake by other APCs. Their activating functions include the induction or boosting of various immune responses, such as the priming of helper and effector cells, production of cytokine and antibodies, and cytotoxicity (136). In contrast, most exosomes produced by epithelial cells in various organs are immunosuppressive, in both health and disease. In health, immunosuppressive exosomes are produced in small quantities by normal epithelia to promote and maintain homeostasis. In disease, such as cancer, as well as during non-disease conditions like pregnancy, high levels of immunosuppressive exosomes are produced, which dysregulate immune responses. These immunosuppressive exosomes assist pregnancy success, but in cancer they promote tumor establishment, growth, and metastasis (124, 137–139). Other disorders involving EVs include pre-eclampsia, endometriosis, polycystic ovarian syndrome, cardiac diseases, rheumatoid arthritis, and allergy (135, 140–143). Mesenchymal stem cell-derived exosomes have been suggested for therapeutic approaches in female reproductive dysfunction (144).

## Role of EVs in endometriosis

### Methodological considerations

Most studies of EVs in endometriosis have focused on characterizing their composition, specifically their protein and non-coding small nucleic acid content (e.g. miRNA and lncRNA). Regarding functional determination, most studies have utilized co-culture experiments to assess the effects of EVs on recipient cells. However, contradictory results have been reported, which could be attributed to the heterogeneity of EV sources and the isolation methodology. EVs, including exosomes, have been isolated from diverse cellular sources and biofluids, including endometriotic epithelium and stromal cells, plasma, serum, and peritoneal fluid. EVs derived from peritoneal fluid comprise a complex mixture, originating not only from endometriotic tissue but also from various other cell types, such as immune cells, fibroblasts and endothelial cells. Consequently, the local extrauterine environment in endometriosis may contain a mixture of immunoactivating and immunosuppressive EVs, consisting of exosomes, microvesicles, and apoptotic bodies. Another important factor to consider is the method of EV isolation: there is significant variation of methods across published reports, ranging from various commercial kits to sucrose gradient ultracentrifugation, with the latter being considered the gold standard for exosome isolation (145, 146). This variation in both sources and isolation methodology complicates comparison of results between studies. Although accumulating evidence supports a role for EVs in endometriosis, the field is still evolving and some proposed mechanisms remain to be consistently validated across independent studies. **Table 6** presents the variation of sources and methods used for EV isolation in the currently published reports.

**Table 6:** Sources and isolation methods of exosomes in endometriosis in cited reports.

Authors	EVs	Source	Isolation method
Khalaj et al., 2019 (149)	EVs Exosomes	Endometriotic lesions Peritoneal fluid Plasma Cell lines	miRCURY exosome isolation kit (Exiqon) Ultrafiltration Ultracentrifugation Verification by TEM*, WB**
Harp et al., 2016 (148)	Exosomes	Supernatant from ECS cultures	Total Exosome Isolation Kit (Invitrogen) Verification by NTA***, TEM
Nazri et al., 2020 (147)	Exosomes	Peritoneal fluid	Exo-spin kit Size-exclusion chromatography Verification by TEM
Zhang et al.,2020 (153)	Exosomes	Serum	Serial Ultracentrifugation Verification by NTA, TEM, WB
Huang et al.,2022 (154)	Exosomes	Supernatant from trypsinized endometriotic tissue	Ultrafiltration Ultracentrifugation Verification by NTA, TEM, WB
Abudula et al., 2022 (152)	Exosomes	Supernatant from cell cultures Vaginal fluid	Ultrafiltration Ultracentrifugation WB
Björk et al., 2024 (45)	Exosomes	Supernatant of short-term endometriotic cultures	Ultrafiltration Sucrose gradient ultracentrifugation Verification by NTA, TEM, IEM****, immunoflow cytometry

*TEM, transmission electron microscopy; **WB, Western blot; ***NTA, nanoparticle tracking analysis; ****IEM, immunoelectron microscopy.

### Studies of protein and non-coding RNA content in EVs in endometriosis

Release of EVs is enhanced in endometriosis, and their composition differs significantly from those isolated from healthy controls. Nazri et al. (147) isolated exosomes from the peritoneal fluid of endometriosis patients and healthy subjects, determining their concentrations across different menstrual cycle phases and disease stages. Notably, exosome concentrations were higher in patients compared to healthy controls. Furthermore, significantly higher levels were produced during the proliferative phase compared to the secretory phase of the menstrual cycle, and in disease stages I/II compared to disease stages III/IV. These findings suggest that EV concentration may serve as a useful biomarker for disease staging. Proteomic analysis further identified five proteins, PRDX1, H2A type 2-C, ANXA2, ITIH4, and tubulin α-chain, that were exclusively present in patients with endometriosis. Harp et al. (148) investigated the miRNA content of exosomes isolated from primary cultures of ectopic and eutopic stromal cells (ESC). The miRNA profiles differed significantly between patients and hormonal phase-matched healthy controls. Treatment with ESC-derived exosomes from patients exerted a pro-angiogenic effect on human umbilical vein endometrial cells (HUVEC), suggesting that these exosomes promote angiogenesis and support the development and survival of endometriotic tissue. Similarly, Khalaj K et al. (149) identified unique miRNA and lncRNA signatures in EVs derived from endometriotic epithelial and endothelial cells, plasma, and peritoneal fluid. When EVs from plasma and peritoneal fluids were taken up by endometriotic epithelial cells, they triggered an upregulation of inflammatory and angiogenic cytokines.

### Endometriotic tissue-derived exosomes carry immunosuppressive signatures

In a recent study (45), we isolated and characterized exosomes secreted by endometriotic lesions. Using a short-term (24h) tissue culture approach, we collected supernatants and isolated exosomes using the established gold-standard protocol (146). We demonstrated that these exosomes carry the NKG2D ligands MICA/B and ULBP1–3, alongside the pro-apoptotic molecules FasL and TRAIL, on their surfaces. These are key signatures of exosome-mediated immune suppression (136). In support of our findings, a dose-dependent upregulation of FasL expression in endometriotic stromal cells following treatment with the macrophage products platelet-derived growth factor (PDGF) and TGFβ has been reported (150). This suggested a Fas-mediated cell death of activated immune cells in endometriosis, which was experimentally proven in our studies (45).

The approach of short-term culture of 24h offered two distinct advantages: 1) a high cell viability, thereby minimizing apoptosis and the subsequent contamination of supernatant with apoptotic blebs and non-secreted vesicles; 2) prevention of contamination with EVs from other sources, through the use of culture supernatants as an EV source, instead of serum or effusions, thus ensuring that only endometriotic lesion-derived exosomes were collected and characterized.

Functional assays revealed that these NKG2D ligand-carrying exosomes downregulate the NKG2D receptor and impair NKG2D-mediated cytotoxicity, thereby disrupting the killing ability of NK cells. Additionally, FasL- and TRAIL-carrying exosomes induced apoptosis in activated peripheral blood mononuclear cells (PBMCs). These results suggest that endometriotic tissue-derived exosomes may eliminate activated immune cells and diminish NK cell potency, providing a mechanistic explanation for the compromised immune surveillance observed in endometriosis (45). Notably, these are recent findings, described for the first time by us, suggesting a possible exosome-mediated mechanism for survival and immune escape of the endometriotic lesions. Thus, they await to be confirmed by other research groups in the field.

### EVs are involved in endometriosis-related inflammation

Many studies imply an inflammatory role of EVs in endometriosis. As reviewed by Giacomini et al. (151), EVs carry pro- and anti-inflammatory mediators that actively participate in disease progression. EVs, which are derived from endometriotic lesions, have been shown to upregulate expression of IL-1β, IL-18, and TNFα (152). Furthermore, Khalaj et al. (149) demonstrated that co-culture with these EVs increases the expression of G-CSF and TNFα in endothelial cells. Zhang et al. (153) reported that miR-22-3p, derived from exosomes of ectopic ESC, can increase the proliferation, migration, and invasion of peritoneal macrophage via the SIRT1/NF-kB signaling pathway. Additionally, lesion-derived exosomal miR-30a-3p has been shown to modulate macrophage polarization (154). Collectively, these findings indicate that EVs from both immune cells and endometriotic stromal cells act in unison to maintain a chronic inflammatory environment at the ectopic sites.

### Bacterial EVs in endometriosis

Emerging evidence suggests that the gut microbiota is altered in patients with endometriosis, and this dysbiosis may modulate immune responses, leading to hormonal imbalances and chronic inflammation (155–157). A recent review by Wagner et al. (158) discussed the potential role of bacteria-derived extracellular vesicles (BEVs) in endometriosis. Drawing on established BEV functions from various bacteria reviewed in (159), the authors hypothesized that BEVs could contribute to endometriosis by inducing cytokine production and macrophage polarization.

The presence of BEVs in endometriosis was first identified via microbiome analysis of peritoneal fluid (160). The microbial composition of BEVs in stages III and IV was found to differ from that of healthy controls, raising questions about their functional role in endometriosis. Unfortunately, the specific bacterial origin, cargo, and functions of these BEVs were not further investigated (160). It should also be noted that the existence of a peritoneal microbiome is still a subject of controversy. It has been suggested that BEVs might play a role in endometriosis (158); however, given the uncertainties mentioned above and the fact that only one, somewhat incomplete, study has been conducted to date (160), it is currently difficult to draw conclusions with certainty regarding the presence or pathogenic role of BEVs in endometriosis.

In summary, EVs in endometriosis encapsulate and carry on their surface a cargo of proteins and non-coding RNAs that mediate key pathogenic mechanisms. Specifically, they: 1) promote angiogenesis and modulate inflammatory responses through the delivery of distinct miRNA and lncRNA profiles; 2) harbor unique structural proteins, which possess potential as diagnostic biomarkers; and 3) facilitate immune evasion by carrying immunosuppressive signature proteins on their surfaces. Collectively, these EV-mediated pathways contribute to the survival and persistence of ectopic endometriotic lesions, offering a mechanistic basis for the clinical manifestations of the disease.

## Discussion

Despite extensive research, the etiology of endometriosis remains elusive, as only a small subset of women experiencing retrograde menstruation develop the disease. However, a substantial body of evidence, detailed in this review, is gradually clarifying the complex factors and mechanisms underlying its pathogenesis. Endometriosis is increasingly viewed as a hormone-driven disorder that is orchestrated by aberrant immune responses that compromise immune surveillance. The inability to clear ectopically seeded endometrial tissue and restore homeostasis instead promotes the implantation, proliferation, and establishment of endometriotic lesions that drive disease initiation and progression.

In the context of endometriosis, a narrative review provides a flexible framework to integrate heterogeneous evidence, encompassing both immunological mechanisms and clinical observations. This approach is particularly valuable in a field characterized by complex, context-dependent immune dysregulation, where findings are often inconsistent and difficult to compare across studies. It facilitates the identification of overarching patterns, discrepancies, knowledge gaps, and emerging hypotheses (161). However, this methodology is inherently limited by the lack of a predefined systematic protocol, which introduces potential selection bias regarding study inclusion and interpretation. The absence of formal quality assessment limits evaluation of evidence strength. Consequently, while this review provides a broad and interpretive overview of immune dysregulation in endometriosis, the conclusions should be considered hypothesis-generating rather than definitive.

The immune profile in endometriosis evolves across disease stages, from impaired immune clearance to a chronic immunosuppressive and fibrotic state. In the early phase, reduced NK-cell cytotoxicity and impaired immune activation prevent effective elimination of ectopic endometrial cells, while increased pro-inflammatory and Th1/Th17-associated cytokines (e.g., IL-1β, IL-6, IL-8, TNFα, IL-17) facilitate their adhesion and implantation. As lesions establish, the immune response shifts toward a mixed inflammatory and regulatory phenotype. This includes a transition from Th1-dominated responses to Th2- and Treg-associated responses, with increasing levels of TGFβ1 and IL-10, expansion of regulatory T cells, and a shift to less cytotoxic NK-cell subsets. This stage is associated with enhanced angiogenesis and initiation of tissue remodeling. In advanced disease, the environment becomes predominantly immunosuppressive and pro-fibrotic, characterized by sustained Th2/Treg activity. Immune evasion is reinforced by exosome-mediated suppression of cytotoxic responses and TGFβ1-driven fibrosis, supporting long-term lesion survival.

**Figure 1:**
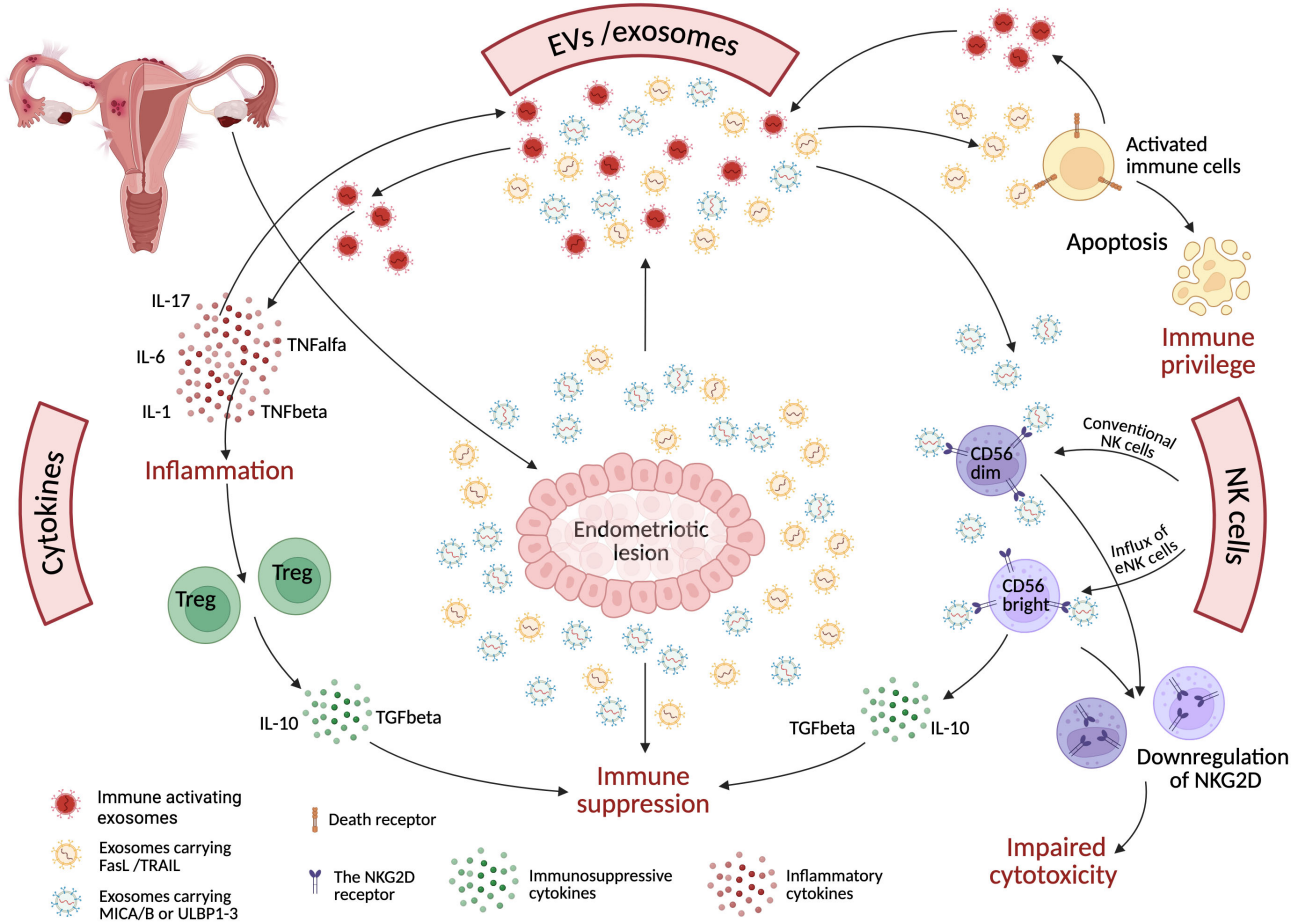
Schematic presentation of mechanisms of protection and survival of ectopic lesions in endometriosis.

This review focuses specifically on NK cells, cytokines, and EVs, including exosomes, which are known as key mediators of immune surveillance and homeostatic regulation. By synthesizing both our own findings and those of others, we propose a conceptual framework (**Figure 1**) that illustrates the mechanisms of immune escape and the protection of endometriotic lesions. In the presented figure and the discussion below, the roles of cytokines and NK-cell dysfunction are based on a synthesis of multiple studies referred to in this review. In contrast, the description of the exosome-mediated immune modulation is based on our recently published results. This mechanism is not yet confirmed by other research groups.

During menstruation, endometrial tissue can be displaced to ectopic sites. This triggers a strong inflammatory response driven by pro-inflammatory cytokines (IL-1β, IL-6, IL-8, TNFα, and TNFβ) released by recruited immune cells and endometriotic lesions. Furthermore, activated macrophages and B cells secrete immunoactivating exosomes that exacerbate this inflammatory milieu. In contrast to the elevated levels of pro-inflammatory cytokines, IFNγ production does not increase as expected, and remains comparable to levels observed in healthy controls (62). This observation suggests a deficient trigger for NK-cell activation. To modulate inflammation, Tregs are recruited to the local microenvironment and activated to secrete the immunosuppressive cytokines TGFβ1 and IL-10. These cytokines not only facilitate immune suppression but also promote the differentiation of naïve T cells into inducible Tregs. Additionally, IL-2 production within the endometriotic lesions elicits local iTreg proliferation (62). Collectively, these mechanisms act in concert to counteract inflammation, thereby establishing and amplifying an immunosuppressive microenvironment at ectopic sites.

As further illustrated in **Figure 1**, endometriotic lesions produce and secrete exosomes, depicted as a “cloud” around the lesions. The intense inflammatory response and the ectopic environment exert significant biological stress on the endometriotic tissue. Such stress induces and enhances the secretion of exosomes bearing FasL/TRAIL and NKG2D ligands (162), which function as potent immunosuppressive mediators (136). Consequently, these lesion-derived immunosuppressive exosomes constitute a protective “shield”, ensuring the survival of the lesions, through two primary mechanisms: 1) exosomes carrying NKG2D ligands downregulate the NKG2D receptor on cytotoxic cells, thereby impairing their lytic capacity; and 2) FasL/TRAIL-bearing exosomes bind to death receptors on activated immune cells and induce their apoptosis (45). Accordingly, lesion-derived immunosuppressive exosomes attenuate the cytotoxic potential of conventional CD56^dim^/CD16^+^ NK cells through NKG2D-receptor downregulation and internalization, while also reducing their numbers via apoptosis. Furthermore, there is an influx of CD56^bright^/CD16^-^ NK cells (38), which intrinsically possess lower cytotoxicity and secrete high levels of TGFβ1 and IL-10, further augmenting local immunosuppression. Taken together, three key factors: 1) exosome-mediated downregulation of the NKG2D receptor; 2) the systemic influx of CD56^bright^/CD16^-^ NK cells; and 3) the absence of a robust IFNγ response, collectively elucidate the characteristic reduction in NK-cell cytotoxicity observed in the peripheral blood and peritoneal fluid of patients with endometriosis. Thus, pro-inflammatory cytokines, immunosuppressive exosomes, and altered NK-cell dynamics act together to establish an immune-privileged microenvironment that facilitates the survival, proliferation, and protection of endometriotic lesions.

The establishment of an immunosuppressive, immune escape-promoting microenvironment mediated by cytokines, exosomes, and NK cells is not unique to endometriosis. The NKG2D receptor–ligand axis is central in various conditions, including pregnancy (122), some viral infections (163–165), autoimmune diseases (166–168) and, most notably, malignancy (162, 169–173). Endometriosis is, to some degree, associated with a moderate risk of developing clear cell and endometrioid ovarian carcinomas (174). The association between endometriosis and risk of other malignancies is weak. However, when we focus on underlying mechanisms, e.g. impaired immune surveillance and sustained chronic inflammation, parallels can be observed between the two disorders (175). Beyond immunological dysfunction, endometriosis shares other features with cancer, including genetic predisposition, angiogenic activity, invasion, proliferation, and the potential for dissemination (174, 176). There are also similarities in microenvironmental interactions and signaling pathways (175, 177). These findings could be an inspiration to test new therapeutic strategies that are currently employed in oncology in the context of endometriosis, which today is mainly treated hormonally and/or surgically (178, 179). Specifically, potential candidates for future clinical evaluation include NK cell-based therapies, including cytokine supplementation or inhibition by monoclonal antibodies; genetically engineered NK cells (180); and antibodies targeting NKG2D ligands (181).

**Figure 2:**
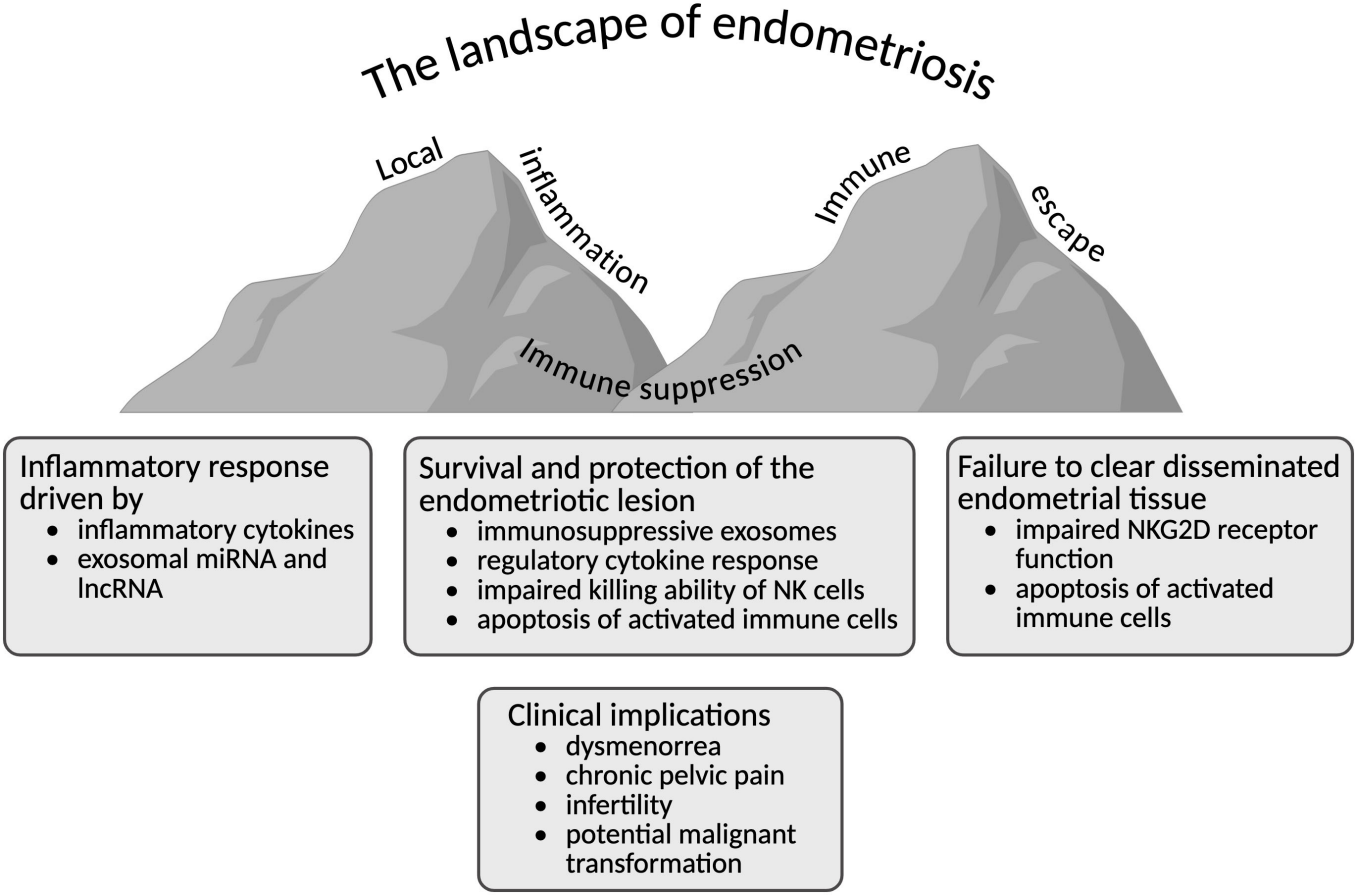
The landscape of endometriosis - summary of the adverse immune reactions that cause the clinical symptoms and define endometriosis as a gynecological disorder rooted in immune dysfunction.

In conclusion, by evaluating the interactions between NK cells, cytokines, and EVs/exosomes, we present compelling evidence that aberrant immunological mechanisms impair immune surveillance and disrupt homeostasis in endometriosis. We provide a mechanistic framework (**Figures 1**, **2**) that elucidates these processes, supporting the concept of endometriosis as a hormonal disorder that is fundamentally rooted in immune dysfunction.
